# Influence of an AQP4 haplotype and sleep duration on early Alzheimer's disease

**DOI:** 10.1002/alz.71540

**Published:** 2026-06-01

**Authors:** Emma L. Palatsides, Stephanie Yiallourou, Dibya Himali, Marina G. Cavuoto, Andrée‐Ann Baril, Qiong Yang, Gina M. Peloso, Joanne Ryan, Georges El Fakhri, Saptaparni Ghosh, Emma Thibault, Charles S. DeCarli, Keith A. Johnson, Alexa S. Beiser, Sudha Seshadri, Jayandra J. Himali, Matthew P. Pase

**Affiliations:** ^1^ Turner Institute for Brain and Mental Health, School of Psychological Sciences, Monash University Melbourne Victoria Australia; ^2^ Framingham Heart Study Framingham Massachusetts USA; ^3^ Department of Neurology Boston University Chobanian & Avedisian School of Medicine Boston Massachusetts USA; ^4^ National Ageing Research Institute Melbourne Victoria Australia; ^5^ Center for Advanced Research in Sleep Medicine Hôpital du Sacré‐Coeur de Montréal, CIUSSS‐NIM Montreal Quebec Canada; ^6^ Department of Medicine Université de Montréal Montreal Quebec Canada; ^7^ Department of Biostatistics Boston University School of Public Health Boston Massachusetts USA; ^8^ School of Public Health and Preventive Medicine Monash University Melbourne Victoria Australia; ^9^ Department of Radiology Yale Biomedical Imaging Institute, Yale School of Medicine New Haven Connecticut USA; ^10^ Department of Radiology Massachusetts General Hospital and Harvard Medical School Boston Massachusetts USA; ^11^ Department of Neurology & Imaging of Dementia and Aging Laboratory University of California, Davis Davis California USA; ^12^ Department of Neurology Massachusetts General Hospital and Harvard Medical School Boston Massachusetts USA; ^13^ Department of Neurology Brigham and Women's Hospital and Harvard Medical School Boston Massachusetts USA; ^14^ Glenn Biggs Institute for Alzheimer's & Neurodegenerative Diseases University of Texas Health Science Center at San Antonio San Antonio Texas USA; ^15^ Department of Neurology University of Texas Health Science Center at San Antonio San Antonio Texas USA; ^16^ Department of Population Health Sciences University of Texas Health Science Center at San Antonio San Antonio Texas USA; ^17^ Graduate School of Biomedical Sciences University of Texas Health Science Center at San Antonio San Antonio Texas USA

**Keywords:** Alzheimer's disease, amyloid, aquaporin‐4, dementia risk factors, framingham heart study, glymphatic system, PET, sleep duration, tau

## Abstract

**INTRODUCTION:**

Aquaporin‐4 (AQP4) is thought to facilitate Alzheimer's disease (AD) protein clearance during sleep. We examined whether *AQP4* genetic variation was associated with AD pathology or modified the association between sleep duration and AD biomarkers.

**METHODS:**

A total of 450 dementia‐free participants (mean age = 58 ± 9.9; women = 54%) from the Framingham Heart Study (FHS) with sleep duration measured by self‐report and amyloid‐*β* (A*β*) and tau burden quantified using positron emission tomography (PET) were analyzed.

**RESULTS:**

*AQP4* was not associated with A*β* or tau burden in the overall sample. However, for participants aged less than 60, minor allele carriers displayed lower regional tau burden compared to homozygote majors. *AQP4* modified the relationship between short sleep (≤6 hours) and medial temporal tau; short sleep duration was associated with higher medial temporal tau in minor allele carriers, while the opposite was observed in homozygote majors.

**DISCUSSION:**

*AQP4* genetic variation may influence early tau accumulation and vulnerability to sleep‐related AD pathology.

## Highlight

- Aquaporin‐4 *(AQP4)* minor allele carriers aged < 60 had lower tau levels than homozygote majors.
- *AQP4* modified the relationship between sleep duration and medial temporal tau.
- The relationship between *AQP4* and tau may depend on age and sleep.

**Abbreviations**
A*β*
: amyloid‐*β*
AD: Alzheimer's disease
AQP4: aquaporin‐4
CSF: cerebrospinal fluid
N3: non‐rapid eye movement stage
SNPs: single nucleotide polymorphisms
PET: positron emission tomography
FHS: Framingham Heart Study
MAF: minor allele frequency (MAF)
HWE: Hardy–Weinberg equilibrium
PAI: Physical Activity Index
PiB: Pittsburgh Compound B
FTP: flortaucipir
FLR: frontal, lateral, and retrosplenial outcome
SAS: Statistical Analysis System
APOE: apolipoprotein E.

## 1 BACKGROUND

The glymphatic system removes waste from the brain, including fibrillated amyloid‐*β* (A*β*) and hyperphosphorylated tau implicated in Alzheimer's disease (AD).^1^, ^2^ Astrocytes, the most abundant glial cell in the brain, are an essential component of this process. They provide structural and functional support to neurons, maintain blood–brain barrier (BBB) integrity, regulate blood flow, and are involved in inflammation signaling in the brain.^3^ An important structure of astrocytes are their perivascular endfeet, which ensheathe cerebral blood vessels, providing a connection between the brain's vasculature and parenchyma.^3^

The process of waste removal from the brain involves the flow of cerebrospinal fluid (CSF) through the brain parenchyma from periarterial spaces, a process facilitated by Aquaporin‐4 (AQP4) channels located primarily on astrocytic endfeet.^1^, ^2^, ^3^, ^4^ AQP4 channels facilitate the movement of water and small molecules across cell membranes.^5^ They are an essential component of the glymphatic system, with genetic deletion of the *AQP4*‐encoding gene shown to reduce glymphatic clearance and increase A*β* and tau accumulation.^6^ In the brain, AQP4 channels are abundantly expressed in the cerebral cortex, cerebellar cortex, ependymal cell layer, and hippocampus.^7^

Sleep plays an essential role in the functioning of the glymphatic system, with the majority of waste clearance thought to occur during slow‐wave sleep (N3 sleep).^8^, ^9^, ^10^ Sleep duration and N3 sleep decline with age and in AD,^11^, ^12^, ^13^ which may impair waste clearance. Likewise, in aging and AD, astrocytes undergo structural remodelling.^14^, ^15^ In AD, AQP4 channels become mislocalized, with reduced perivascular polarization to endfeet and increased localization to astrocytic cell bodies, particularly in the frontal cortex.^16^, ^17^, ^18^ AQP4 expression levels naturally increase with age, but can be up or down regulated in AD.^16^, ^17^, ^19^, ^20^ Together, these changes to sleep and AQP4 channels may alter waste clearance from the brain. Thus, glymphatic efficiency depends on the duration of physiological states that support waste clearance (e.g., N3 sleep) and the integrity of its underlying mechanisms, including AQP4 localization and expression.

Genetic variation at specific *AQP4* single nucleotide polymorphisms (SNPs) has been linked to differences in AQP4 expression levels and the intensity of slow waves in N3 sleep. ^21^, ^22^, ^23^ In particular, minor allele carriers at a specific *AQP4* haplotype have been associated with a lower expression of AQP4 channels using in vitro luciferase assays,^21^, ^22^ yet more slow‐wave energy and activity.^23^ These findings have been interpreted to suggest that increased slow‐wave activity may partially compensate for reduced cannel expression, potentially supporting glymphatic clearance efficiency.^23^ Consistent with this contention, we have recently demonstrated that carrying the minor allele is associated with better memory, larger hippocampal volumes, lower amounts of brain free water, and lower dementia risk compared to homozygote majors.^24^

Despite the role of AQP4 water channels in brain fluid flow, little is known about whether *AQP4* genetic variation is related to early brain changes associated with AD. Some emerging evidence has linked *AQP4* to A*β* burden and sleep.^25^, ^26^ One study found shorter self‐report sleep duration was associated with higher brain A*β* burden in minor allele carriers at rs72878776 and rs491148.^25^ However, the relationship between *AQP4* with tau pathology and sleep remains unexplored.

**RESEARCH IN CONTEXT**

1. **Systematic review**: The authors searched for literature using databases (e.g., google scholar, PubMed). There have been few studies that have explored the association between aquaporin‐4 (*AQP4)* and Alzheimer's disease (AD) pathology. These were cited within the paper.
2. **Interpretation**: We found that minor allele carriers at an *AQP4* haplotype aged less than 60 years had significantly lower tau deposition compared to homozygote majors. In individuals with short sleep, minor allele carriers tended to have greater tau deposition in the medial temporal lobe whereas homozygote majors had lower tau deposition in this region. These findings highlight that the associations between *AQP4* and AD appear context‐dependent, whereby carrying the minor allele may be protective at younger ages and under optimal sleep conditions.
3. **Future directions**: Future studies should explore whether the findings from this study replicate within different samples and with the use of objective measures of sleep.

We aimed to examine the association between *AQP4* and early pathological hallmarks of AD using A*β* and tau positron emission tomography (PET) imaging. Using a previously described *AQP4* haplotype to determine genetic variation,^23^, ^24^ we hypothesized that minor allele carriers would have lower levels of A*β* and tau, due to the proposed compensatory mechanism. We also explored whether these associations were moderated by age or sex. Since both sleep and AQP4 water channels are involved in glymphatic clearance, our second aim was to examine if the associations between self‐report sleep duration and AD biomarkers were modified by *AQP4*. We hypothesized that the association between short sleep duration and higher amounts of A*β* and tau would be strongest in the minor allele carrier group compared to homozygote majors, as the combination of insufficient sleep time and genetically associated reductions in AQP4 expression may limit glymphatic clearance capacity.

## 2 METHODS

### 2.1 Participants

Participants were from the multigenerational, prospective community‐based, Framingham Heart Study (FHS). We included participants from the FHS Offspring and Generation 3 cohorts who had both genetic and PET data available. The Offspring cohort was enrolled in 1971 and included 5124 participants who were the biological children, adoptive children, or spouses of the Original cohort.^27^ A third generation of participants were recruited in 2002 and included 4095 children of the Offspring cohort.^28^ Participation in FHS involves completing regular examination cycles and participants are continuously monitored for medical events such as myocardial infarction, stroke, and dementia.^29^

The PET sample was a subset of the Offspring and Generation 3 cohorts. To be eligible for PET scans, FHS participants had to be 30 years or older, have previously completed a brain magnetic resonance imaging (MRI) scan as part of their participation, and be without dementia, stroke, or another significant neurological condition. All participants provided written informed consent, and ethical approval was obtained from the Institutional Review Boards at Boston University School of Medicine and the Human Research Ethics Committee at Monash University. The study was conducted in line with the Declaration of Helsinki.

### 2.2 Genotyping of AQP4

Affymetrix GeneChip Human Mapping 500K Array and the 50K Human Gene Focused Panel was used to genotype participant DNA from peripheral blood samples at the Affymetrix Research Services Laboratory in Santa Clara, California, USA. Samples were excluded if a participant call rate was less than 97%, a per‐subject heterozygosity was ± 5 SD from the mean, or a per‐subject number of Mendelian errors was greater than 165 (99th quantile). 8481 individuals had 433,510 SNPs genotyped that passed these quality‐control measures. The Bayesian Robust Linear Model with Mahalanobis distance algorithm was used for allele calling.^30^ A subset of 425,173 SNPs were used in a principal‐components analysis to evaluate the population structure (infer axes of variation) with a minor allele frequency (MAF) ≥ 0.01, Hardy–Weinberg equilibrium (HWE) *p* ≥ 10−6, and call rate ≥ 0.95.

We used an *AQP4* haplotype to determine genetic variation. Three (rs335931, rs335929, and rs16942851) out of the eight SNPs that comprise the haplotype (rs162007, rs162008, rs63514, rs455671, rs335931, rs335930, rs335929, and rs16942851) were analyzed because they are in high linkage disequilibrium with all other SNPs within the haplotype.^23^ Since only a small percentage of participants were homozygote for the minor allele (1.78%), we classified participants into one of the following two *AQP4* groups: homozygote major (no minor alleles); minor allele carrier [heterozygote (one minor allele) or homozygote minor (two minor alleles)].

### 2.3 PET imaging

PET imaging was completed between 2015 and 2022 using C‐Pittsburgh Compound B (PiB) for A*β* and flortaucipir (FTP) for neurofibrillary tangle tau. The images were completed across two scanners: Siemens ECAT HR+ (3D mode; 63 image planes; 15.2 cm axial field of view; 5.6 mm transaxial resolution; and 2.4 mm slice interval) and 5‐Ring Discovery GE.^31^ Briefly, the PiB images required a 10 to 15 mCi bolus injection and the dynamic images were acquired over a 60 min period. The FTP images required a 9 to 11 mCi bolus injection and were acquired across an 80‐to‐100‐min period. The PiB and FTP images tended to be completed on the same day and co‐registered to a structural T1 weighted brain MRI using SPM8 and FreeSurfer v6.0 to obtain the brain regions of interest. PiB retention was expressed as a distribution volume ratio whereas FTP retention was expressed as a standardized uptake value. Both PiB and FTP data collected using the GE camera were smoothed using a 6 mm Gaussian filter for harmonization. Additionally, PiB and FTP retention used the cerebellar cortex as a reference region. No partial volume correction was applied due to the relatively young age of participants and minimal atrophy.

For A*β* imaging, we included a summary measure of the weighted average of the following frontal, lateral, and retrosplenial (FLR) brain regions: inferior temporal, medial temporal, superior temporal, transverse temporal, supramarginal, inferior parietal, superior parietal, insula, lateral orbitofrontal, pars orbitalis, pars triangularis, pars opercularis, caudal middle frontal, rostral middle frontal, superior frontal, medial orbitofrontal, rostral anterior cingulate, caudal anterior cingulate, posterior cingulate, precuneus, and isthmus cingulate. For tau, we calculated tracer uptake in the following regions: entorhinal, rhinal, inferior temporal, and fusiform regions. We also calculated a medial temporal lobe summary region composed of the hippocampus, amygdala, and entorhinal regions. All brain regions were included based on previous research highlighting their association with A*β* or tau in AD.^32^, ^33^, [Fig alz71540-fig-0001]

### 2.4 Self‐reported sleep duration

For Aim 2, self‐reported sleep duration was measured using the Physical Activity Index (PAI) questionnaire. We included Offspring participants at their nineth clinical examination (2011–2014) and Generation 3 participants at their third clinical examination (2016–2019) who completed this questionnaire. Participants were asked the “number of hours that you typically sleep” and were categorized as having “short sleep” if they had less than or equal to 6 hours of sleep and “normal sleep” if they had greater than 6 to less than 9 hours of sleep (reference group).^34^ We excluded long sleepers (≥9 hours) so that the reference group comprised only of individuals with normal sleep duration. Only 5.6% of participants belonged to the long sleep duration category, and thus, we were underpowered to study this sleep phenotype.

### 2.5 Statistical analyses

For Aim 1, linear regressions were performed to examine the associations between *AQP4* genetic variation and the PET outcomes. These analyses were adjusted for age, age squared, sex, apolipoprotein E *(APOE*, ε4 carrier vs. noncarrier), and PET camera type. Age squared was included as an adjustment because age has a nonlinear relationship with A*β* and tau burden. Age (< 60 vs. ≥ 60 years) and sex were included as interaction terms for these analyses.

For Aim 2, a linear regression model was conducted to examine whether *AQP4* genetic variation modified the relationship between sleep duration (short sleep vs. normal sleep duration) and the PET outcomes. These analyses were adjusted for age, age squared, sex, *APOE* (ε4 carrier vs. noncarrier), PET camera type, and the time interval between self‐reported sleep duration measurement and PET imaging. All results were considered statistically significant if *p *< 0.05. All analyses were completed using the Statistical Analysis System (SAS) software v9.4.

## 3 RESULTS

### 3.1 Sample characteristics

**FIGURE 1 alz71540-fig-0001:**
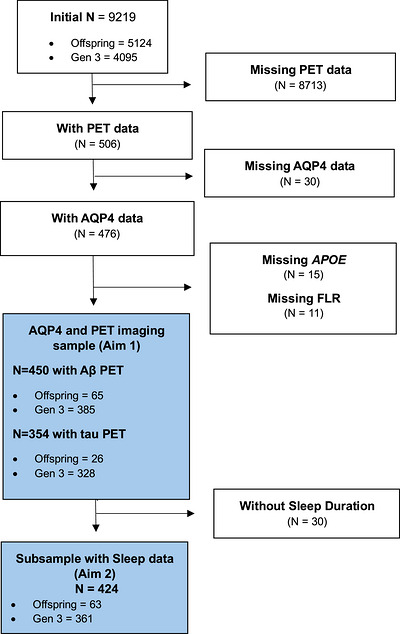
Sample selection diagram. A*β*, amyloid‐*β*; APOE, apolipoprotein E; FLR, frontal, lateral, and retrosplenial outcome; PET, positron emission tomography.

See Figure 1 for sample selection information. Characteristics for the Aim 1 analysis sample (*AQP4* and PET burden; *N* = 450) are presented in Table 1. 68% of participants were homozygote major and 32% were minor allele carriers. The mean age of participants was 58 years; 54% were women. A large subset of this sample (94%; *N* = 424) was used for Aim 2 (*AQP4* and sleep duration interaction on PET burden, see Table S1 for sample characteristics), with exclusions due to not completing the PAI questionnaire. In brief, 114 (27%) were short sleepers (≤6 hours); 75% of these were homozygote majors and 25% were minor allele carriers.

**TABLE 1:** Overall sample characteristics

*AQP4* haplotype group	Homozygote major (no minor alleles)	Minor allele carriers	Overall sample
N	308	142	450
Age, years	57.6 (9.9)	57.4 (9.0)	57.6 (9.6)
Sex, women, *n* (%)	167 (54.2%)	76 (53.5%)	243 (54.0%)
Level of education, *n* (%)			
No high school degree	1 (0.3%)	0 (0.0%)	1 (0.2%)
High school degree	28 (9.1%)	14 (9.9%)	42 (9.3%)
Some college	62 (20.1%)	44 (31.0%)	106 (23.6%)
College degree	217 (70.5%)	84 (59.2%)	301 (66.9%)
*APOE* e4 carrier, *n* (%)	74 (24.0%)	31 (21.8%)	105 (23.3%)
PET camera A*β*			
Discovery GE smoothed	97 (31.5%)	46 (32.4%)	143 (31.8%)
HR+	211 (68.5%)	96 (67.6%)	307 (68.2%)
PET camera tau			
Discovery GE smoothed	58 (18.8%)	30 (21.1%)	88 (19.6%)
HR+	182 (59.1%)	86 (60.6%)	268 (59.6%)
PET outcomes			
FLR A*β*	1.08 (0.11)	1.08 (0.11)	1.08 (0.11)
Entorhinal tau	1.06 (0.09)	1.05 (0.11)	1.06 (0.10)
Rhinal tau	1.10 (0.09)	1.09 (0.12)	1.10 (0.10)
Inferior temporal tau	1.15 (0.08)	1.14 (0.09)	1.15 (0.08)
Fusiform tau	1.15 (0.07)	1.14 (0.09)	1.14 (0.08)
Medial temporal lobe tau	1.10 (0.08)	1.10 (0.09)	1.10 (0.08)

*Note*: Data are mean (SD) unless specified otherwise.

Abbreviations: A*β*, amyloid‐*β*; APOE, apolipoprotein E; FLR, frontal, lateral, and retrosplenial outcome; PET, positron emission tomography.

### 3.2 Association between AQP4 and PET outcomes

The associations between *AQP4* and all PET outcomes were not significant (Table 2).

**TABLE 2:** Association between *AQP4* and PET outcomes with age and sex interactions.

		Main effects		Interactions	
Parameter	*N*	*β* (95% CI)	*p‐*value	*p‐*value (*AQP4 ×* age)	*p value (AQP4 ×* sex)
FLR A*β* ^*^	450			0.546	0.764
Homozygote major	308	REF			
Minor allele carriers	142	0.005 (−0.012, 0.021)	0.585		
Entorhinal tau	354			**0.040**	0.578
Homozygote major	238	REF			
Minor allele carriers	116	−0.005 (−0.026, 0.017)	0.672		
Rhinal tau	343			**0.013**	0.718
Homozygote major	227	REF			
Minor allele carriers	116	−0.013 (−0.036, 0.009)	0.247		
Inferior temporal tau	354			**0.025**	0.932
Homozygote major	238	REF			
Minor allele carriers	116	−0.008 (−0.025, 0.009)	0.382		
Fusiform tau	354			**0.008**	0.615
Homozygote major	238	REF			
Minor allele carriers	116	−0.007 (−0.024, 0.009)	0.396		
**0.011**Middle temporal lobe tau	354			**0.011**	0.994
Homozygote major	238	REF			
Minor allele carriers	116	−0.007 (−0.023, 0.009)	0.391		

*Note*: All analyses were adjusted for age, age squared, sex, *APOE* (ε4 carrier vs. non‐carrier), and camera. Bold indicates statistical significance, *p *< 0.05.

* Values were natural log transformed.

Abbreviations: Aβ, amyloid‐β; *AQP4*, aquaporin‐4; CI, confidence interval; FLR, frontal, lateral, and retrosplenial outcome; PET, positron emission tomography; REF, reference.

### 3.3 Interactions with age and sex

**FIGURE 2:**
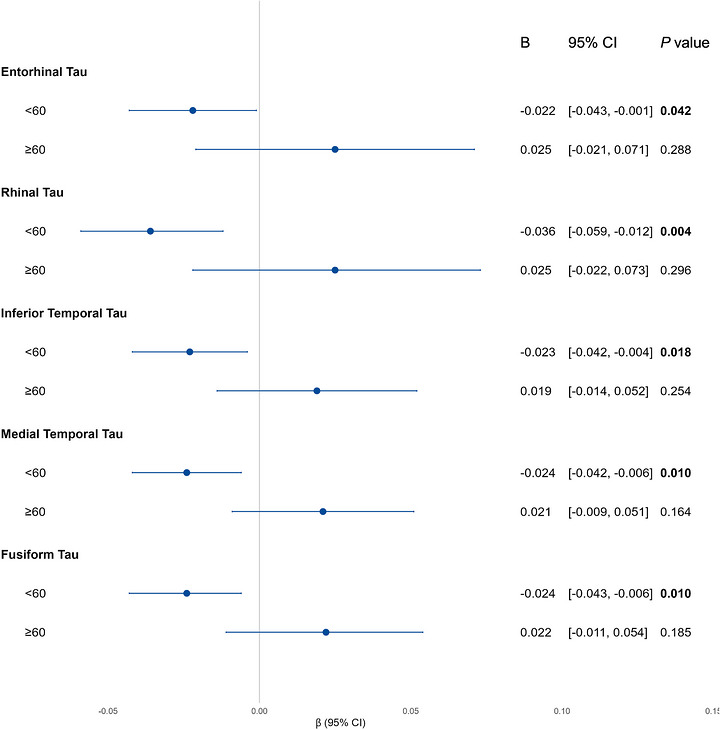
Age stratification for the association between *AQP4* and tau burden. The results show estimates for minor allele carriers relative to homozygote majors (reference group). All analyses were adjusted for age, age squared, sex, *APOE* (ε4 carrier vs. noncarrier), and camera. Bold indicates statistical significance, *p* < 0.05. *AQP4*, aquaporin‐4; APOE, apolipoprotein E; CI, confidence interval.

There were significant interactions between age and *AQP4* for all of the tau PET outcomes (Table 2). In the younger age group (< 60 years), minor allele carriers had lower tau burden compared to homozygote majors (Figure 2). In the older age group (≥ 60 years), the point estimates were in the opposite direction, but confidence intervals were wide and included the null. No other interactions were observed.

### 3.4 Modifying effect of AQP4 on the relationship between sleep duration and PET outcomes

**FIGURE 3:**
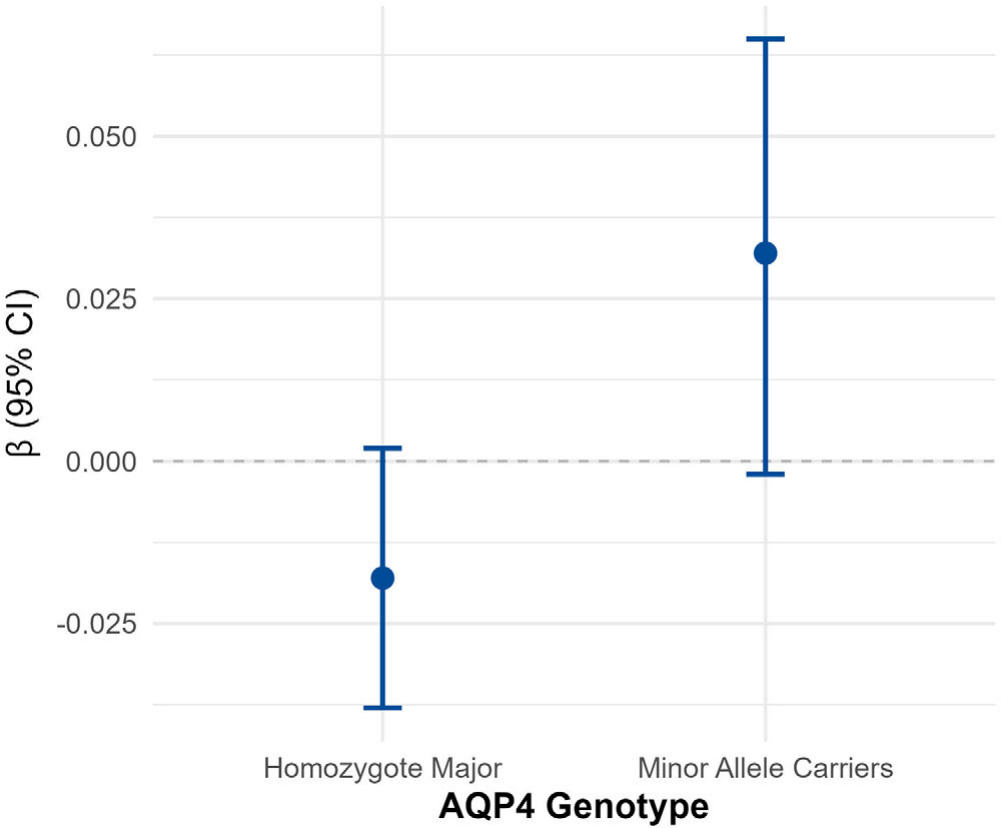
Modifying effect of *AQP4* on the relationship between sleep duration and medial temporal lobe tau. Figure shows the effect of short sleep duration (≤ 6 hours) versus normal sleep duration (> 6 to < 9 hours) on medial temporal lobe tau stratified by *AQP4* status. All analyses were adjusted for age, age squared, sex, *APOE* (ε4 carrier vs. non‐carrier), camera, and time between sleep assessment and PET imaging; Bold indicates statistical significance, *p* < 0.05; The effect for homozygote majors was (*β*[95% CI], −0.018[−0.038, 0.002],* p *= 0.084, *N* = 222). The effect for minor allele carriers was (*β*[95% CI], 0.032[−0.002, 0.065],* p *= 0.061, *N* = 109). *AQP4*, aquaporin‐4; APOE, apolipoprotein E; CI, confidence interval; positron emission tomography.

The associations between sleep duration and the PET outcomes are reported elsewhere^35^ (see Table S2 for a summary of results in this analysis sample). We found that *AQP4* modified the relationship between sleep duration and medial temporal lobe tau (Figure 3), such that *AQP4* minor allele carriers with short sleep duration tended to have a higher amount of tau in the medial temporal lobe whereas homozygote majors with short sleep duration had lower amounts of tau in this same region. *AQP4* did not modify the relationship between sleep duration and any other PET outcome (Table S2).

## 4 DISCUSSION

In this study, we identified context‐dependent associations between *AQP4* genetic variation, tau pathology, and sleep duration. Although *AQP4* was not associated with A*β* or tau burden in the overall sample, two consistent patterns emerged. First, among adults aged less than 60 years, those carrying the *AQP4* minor allele exhibited lower tau burden compared to homozygote majors, suggesting a potential protective effect earlier in life. Second, *AQP4* modified the relationship between sleep duration and tau, such that minor allele carriers with short sleep showed higher tau levels in the medial temporal lobe, whereas homozygote majors with short sleep showed lower tau levels in this region. No such interactions were observed for A*β*. Together, these findings suggest a complex relationship between *AQP4* and AD pathology, with age and sleep dependent effects on tau accumulation.

The first aim of the present study was to examine the association between an *AQP4* haplotype and PET imaging markers of AD. Overall, there were no significant associations between *AQP4* and the PET outcomes. These findings are consistent with a previous study^25^ that reported no association between *AQP4* and A*β* burden in 222 participants aged greater than 60 years. We extend these findings by using different *AQP4* SNPs and tau imaging, showing no direct association between *AQP4* and tau.

Importantly, we identified that the associations between *AQP4* and tau were modified by age. Minor allele carriers aged less than 60 years had lower tau PET tracer uptake compared to homozygote majors across brain regions. In participants aged 60 years or older, estimates were less precise and did not provide clear evidence of an association. Accordingly, the age interaction should be interpreted as evidence that the association between *AQP4* and tau differs across age groups, with the clearest signal observed in younger participants. The potential mechanisms underlying this protective effect of the *AQP4* minor allele at younger ages are unclear. Carriage of the minor allele has been associated with increased slow‐wave energy and activity,^23^ which promotes glymphatic clearance of metabolic waste, including tau.^36^ This could confer resilience to tau accumulation during midlife. However, in normal aging, sleep quality declines, and those aged less than 60 years experience a marked reduction,^37^ potentially attenuating this protective effect at older ages. In addition, vascular changes, which become more common with aging,^38^, ^39^ can affect AQP4 channels. In mouse models, the quantity of AQP4 channels is reduced in brain regions with vascular damage,^40^ which may further compromise clearance mechanisms. Together, these findings suggest that *AQP4* may influence tau accumulation during midlife, prior to the onset of clinical symptoms of AD dementia.

Unlike tau, we did not observe a significant age interaction for the association between *AQP4* and A*β*. Although A*β* typically accumulates prior to tau in AD, the mechanisms underlying this differential association remain unclear and require further investigation.

The second aim of our study was to investigate whether the *AQP4* haplotype modified the relationship between sleep duration and AD biomarkers. Prior work found that different *AQP4* SNPs than those included in our study moderated the relationship between self‐report sleep duration and A*β* burden.^25^ Short sleep was associated with higher A*β* burden in those who carried the minor allele at some SNPs.^25^ We found that short sleep duration (relative to normal sleep duration) was associated with greater medial temporal tau deposition in minor allele carriers and lower tau in homozygote majors. This aligns with our hypothesis that, under conditions that stress the glymphatic system, such as insufficient sleep, the *AQP4* genotype that lowers AQP4 expression (minor allele) may increase vulnerability to AD. In other words, in the context of short sleep, which limits the opportunity for glymphatic clearance, minor allele carriers may be unable to compensate for reduced channel expression.

In our previous work, we found that minor allele carriers had larger hippocampal volumes, better verbal episodic memory, and lower dementia risk compared to homozygote majors.^24^ The finding that medial temporal lobe tau differs by *AQP4* is therefore consistent with our previous work, given that medial temporal tau, hippocampal atrophy, and episodic memory deficits are signatures of AD dementia. However, prior work in this cohort indicates that medial temporal lobe tau burden is not associated with memory performance in midlife,^41^ suggesting that the regional tau differences observed here are likely to reflect early pathological variation rather than clinical disease expression.

We also found that homozygote majors reporting short sleep duration had lower medial temporal lobe tau compared to homozygote majors reporting normal sleep duration. The direction of this association was unexpected and does not align with prevailing models linking insufficient sleep to increased pathological burden.^42^ The mechanisms underlying this finding are not yet clear and replication in independent samples will be important.

### 4.1 Strengths, limitations, and future directions

Strengths of our study include the relatively large number of participants who underwent PET scans for both A*β* and tau. The community‐based sample and relatively young age of participants are also strengths, allowing us to capture early elevations in AD pathology in a community sample.

Use of a subjective measure of sleep is a limitation of our study. Although self‐report may better capture habitual sleep durations, subjective measures are subject to recall bias. Future studies using objective measures of sleep such as polysomnography, which provides an estimate of N3 sleep duration and delta power, may be more relevant to glymphatic clearance than overall sleep duration. Additionally, incorporating objective circadian phase and timing may help clarify whether sleep–wake alignment is associated with *AQP4* and subsequent neurodegenerative risk.

Importantly, the functional consequences of altered AQP4 expression in aging and AD are complex and not fully understood. Although changes in AQP4 expression and localization have been reported in AD,^16^, ^17^, ^18^ the present study does not allow us to determine how *AQP4* influences channel distribution, astrocytic responses, or glymphatic function directly. Accordingly, our findings should be interpreted as systems‐level genetic associations rather than direct evidence of specific cellular mechanisms.

Future research should examine whether *AQP4* relates to alterations in cellular distribution or polarization and astrocytic regulatory responses. Such work would help clarify the biological pathways underlying the associations observed in minor allele carriers.

### 4.2 Implications

The observed age interaction for the association between *AQP4* and tau suggests that *AQP4* genetic effects on tau accumulation may be most apparent during the early, preclinical stages of AD. The absence of this effect in older participants indicates that age‐related processes may attenuate or obscure these associations later in life. This underscores the importance of considering age when evaluating genetic influences on AD biomarkers.

The finding that *AQP4* modified the association between short sleep and tau burden suggests that the neurobiological consequences of insufficient sleep may differ according to *AQP4* genetic variation. This observation may help explain the heterogeneity across studies linking sleep and AD.^43^, ^44^, ^45^, ^46^ In addition, they support the concept that behavioral risk factors for AD may vary across individuals. If replicated longitudinally, these results may suggest that particular sleep strategies could be relevant for individuals carrying specific *AQP4* genetic variants.

## 5 CONCLUSION

Poor sleep has been associated with poorer brain health,^12^ although findings differ across studies.^43^, ^44^, ^45^, ^46^ This variability may reflect that some individuals are more susceptible to the neurobiological consequences of poor sleep than others. Our study suggests that genetic variation, such as differences in the *AQP4* genotype, may partly account for this differential vulnerability to short sleep. Further research into the complex interactions between behavioral and genetic risk factors may help inform more personalized prevention strategies. For example, short sleep may represent a behavioral risk factor for AD for minor allele carriers at the *AQP4* haplotype. Another important finding from our study was that minor allele carriers aged less than 60 years had significantly lower tau deposition across all regions examined compared to homozygote majors. Collectively, these findings reinforce the context‐dependent associations between *AQP4* and AD. The findings suggest that the protective effect of the minor allele may be most apparent in individuals with optimal sleep duration and at younger ages, and that this advantage may diminish with aging or even reverse with conditions that stress the glymphatic system.

## CONFLICT OF INTEREST STATEMENT

The authors declare no conflicts of interest. Author disclosures are available in the Supporting Information.

## CONSENT STATEMENT

All participants provided informed consent.

## DISCLOSURES

The authors have no disclosures to report.

## Supporting information



Supporting Information

Supporting Information
