# Supplementary material for: Influence of an AQP4 haplotype and sleep duration on early Alzheimer's disease
Source: Alzheimers Dement. 2026 Jun 1;22(6):e71540. doi: 10.1002/alz.71540 (PMC13239115; doi:10.1002/alz.71540)
Supplement: Supplementary file 2 — Supporting Information [file ALZ-22-e71540-s002.docx]

**Supplementary Materials for
Palatsides, Yiallourou, Himali et al.** **Influence of an AQP4 haplotype and sleep duration on early Alzheimer’s disease**

**Contents**

Supplementary Table 1. Characteristics of the Sleep Sub-Sample………………………………..2
Supplementary Table 2. Association Between Sleep Duration and PET Outcomes with *AQP4* Interaction*……………………………………………………..*…………………………………………4

| **Supplementary Table 1.** Characteristics of the Sleep Sub-Sample  **Total** =   - Offspring = - Gen 3 =   **With missing covariates** =   - Offspring = - Gen 3 =   **Without rare *AQP4*** (N = )  **With rare *AQP4*** =   - Offspring = - Gen 3 =   **With known dementia status** (N = )  **With other neurological conditions**=   - Offspring = - Gen 3 =   **Without dementia** (N = )  **With unknown dementia status** =   - Offspring = - Gen 3 =   **Without other neurological conditions** (N = )  **With dementia** =   - Offspring = - Gen 3 =   **Aged 45 or over** (N = )  **Age < 45 years** =   - Offspring = - Gen 3 =   **PET data available** (N = )  **Without PET =**   - Offspring = - Gen 3 =   **Genetic data available** (N = )  **Missing genetic data** =   - Offspring = - Gen 3 = | | | |
| --- | --- | --- | --- |
| Sleep duration | Normal (>6 to <9 hours) | Short (≤6 hours) | Overall sample |
| N | 310 | 114 | 424 |
| Age, years | 54.5 (8.4) | 53.9 (8.7) | 54.4 (8.4) |
| Sex, women, n (%) | 172 (55.5%) | 59 (51.8%) | 231 (54.5%) |
| Level of Education, n (%)  No high school degree | 1 (0.3%) | 0 (0.0%) | 1 (0.2%) |
| High school degree | 21 (6.8%) | 16 (14.0%) | 37 (8.7%) |
| Some college | 66 (21.3%) | 33 (29.0%) | 99 (23.4%) |
| College degree | 222 (71.6%) | 65 (57.0%) | 287 (67.7%) |
| *APOE* e4 carrier, n (%) | 80 (25.8%) | 22 (19.3%) | 102 (24.1%) |
| *AQP4*  Homozygote major, n (%) | 204 (65.8%) | 85 (74.6%) | 289 (68.2%) |
| Minor allele carriers, n (%) | 106 (34.2%) | 29 (25.4%) | 135 (31.8%) |
| PET Camera Aβ  Discovery GE smoothed | 102 (32.9%) | 36 (31.6%) | 138 (32.5%) |
| HR+ | 208 (67.10%) | 78 (68.4%) | 286 (67.5%) |
| PET Camera Tau  Discovery GE smoothed | 60 (19.4%) | 24 (21.1%) | 84 (19.8%) |
| HR+ | 179 (57.7%) | 69 (60.5%) | 248 (58.5%) |
| PET outcomes  FLR Aβ | 1.09 (0.12) | 1.08 (0.09) | 1.08 (0.11) |
| Entorhinal Tau | 1.06 (0.10) | 1.05 (0.09) | 1.06 (0.10) |
| Rhinal Tau | 1.10 (0.11) | 1.09 (0.10) | 1.10 (0.10) |
| Inferior Temporal Tau | 1.15 (0.09) | 1.13 (0.07) | 1.15 (0.08) |
| Fusiform Tau | 1.15 (0.08) | 1.13 (0.07) | 1.14 (0.08) |
| Medial Temporal Lobe Tau | 1.10 (0.08) | 1.09 (0.08) | 1.10 (0.08) |
| Sleep duration | 7.4 (0.5) | 5.7 (0.6) | 7.0 (0.9) |
| Time between sleep assessment and Aβ PET | 3.3 (2.9) | 2.8 (2.9) | 3.1 (2.9) |
| Time between sleep assessment and tau PET | 2.6 (2.7) | 2.3 (2.7) | 2.6 (2.7) |
| NOTE. Data are mean (SD) unless specified otherwise. Abbreviations: FLR=frontal, lateral, and retrosplenial outcome; Aβ=Amyloid-β; PET=Positron Emission Tomography. | | | |

| **Supplementary Table 2.** Association between Sleep duration and PET Outcomes with *AQP4* Interaction | | | | |
| --- | --- | --- | --- | --- |
|  | **N** | **β (95% CI)** | ***P* value (main effects)** | ***P* value (AQP4 interaction)** |
| FLR Aβ* | 424 |  |  |  |
| Normal sleep | 310 | REF |  |  |
| Short sleep | 114 | -0.001 (-0.019, 0.016) | 0.874 | 0.614 |
|  |  |  |  |  |
| Entorhinal Tau | 331 |  |  |  |
| Normal sleep | 238 | REF |  |  |
| Short Sleep | 93 | -0.005 (-0.027, 0.018) | 0.688 | 0.398 |
|  |  |  |  |  |
| Rhinal Tau | 320 |  |  |  |
| Normal sleep | 230 | REF |  |  |
| Short Sleep | 90 | -0.010 (-0.034, 0.015) | 0.440 | 0.666 |
|  |  |  |  |  |
| Inferior Temporal Tau | 331 |  |  |  |
| Normal sleep | 238 | REF |  |  |
| Short sleep | 93 | -0.014 (-0.032, 0.004) | 0.133 | 0.266 |
|  |  |  |  |  |
| Medial Temporal Tau | 331 |  |  |  |
| Normal sleep | 238 | REF |  |  |
| Short sleep | 93 | -0.003 (-0.020, 0.014) | 0.746 | **0.015** |
|  |  |  |  |  |
| Fusiform Tau | 331 |  |  |  |
| Normal sleep | 238 | REF |  |  |
| Short sleep | 93 | -0.015 (-0.033, 0.002) | 0.089 | 0.810 |
| NOTE. All analyses were adjusted for age, aged squared, sex, *APOE* (ε4 carrier versus non-carrier), camera, and time between sleep assessment and PET imaging; Bold indicates statistical significance, *P*<.05; *values were natural log transformed; Abbreviations: FLR=frontal, lateral, and retrosplenial outcome; Aβ=Amyloid-β; CI=confidence interval; PET=Positron Emission Tomography. | | | | |
